# Kidney Diseases and Their Impact on the Saudi Economy: The Role of Pediatric Nephrology in Long-Term National Growth, Challenges, and Solutions

**DOI:** 10.7759/cureus.102577

**Published:** 2026-01-29

**Authors:** Mugahid Elhag Elamin, Wafa Daw, Khalid Mohammed, Ameerah Elhaj Alamin, Mawaheb Alameen, Abdelfatah Farah, Reem Mohamed, Majed Aloufi

**Affiliations:** 1 Pediatric Nephrology Department, Prince Sultan Military Medical City, Riyadh, SAU; 2 Ophthalmology Department, Prince Sultan Military Medical City, Riyadh, SAU; 3 Clinical Pharmacy and Business Management, Baxter Company Limited, Riyadh, SAU; 4 Department of Histopathology and Cytology, Saudi German Health, Middle East Healthcare Co., Riyadh, SAU; 5 Pharmacy, Elrazi University, Khartoum, SDN; 6 Education, Arab Bureau of Education for the Gulf States, Riyadh, SAU

**Keywords:** chronic kidney disease, health economics, kidney transplantation, pediatric nephrology, saudi vision 2030

## Abstract

Kidney disease in children is a major health issue in Saudi Arabia. Many cases of chronic kidney disease (CKD) and end-stage kidney disease (ESKD) in children are caused by congenital anomalies. Early dialysis and long-term care are expensive for the national healthcare system. This review examines the economic impact of pediatric kidney diseases and explores how investing in pediatric nephrology could reduce long-term health care costs and support national economic growth. This is a narrative review of published studies on pediatric kidney disease in Saudi Arabian patients. The aim of this review is to examine the health costs and economic impact of dialysis and transplantation. Detecting kidney disease early, treating it quickly, and investing in pediatric transplants can greatly reduce healthcare costs over time. Affordable options such as peritoneal dialysis and preventive care help lower the strain on government budgets. When children are healthier, they do better in school, become more productive adults, and are more likely to join the workforce. Growing pediatric kidney care can also attract medical tourism, encourage healthcare entrepreneurship, and promote research and innovation. Pediatric nephrology brings important health and economic benefits to Saudi Arabia. Detecting kidney disease early, growing transplant programs, using cost-effective treatments, and supporting research are all key to lessening the impact of kidney disease in children. To achieve these benefits and support Saudi Vision 2030, everyone involved should work together and act quickly. It is imperative to enhance early detection initiatives, increase investment in transplant infrastructure, expand financial support for pediatric kidney research, and raise public awareness.

## Introduction and background

Pediatric kidney disease is a serious public health issue in Saudi Arabia. Many cases of chronic kidney disease (CKD) in children are caused by congenital anomalies of the kidney and urinary tract (CAKUT), which are the main cause of end-stage kidney disease (ESKD) in children worldwide [1,2]. Many of these children need dialysis early, sometimes even as newborns, which leads to high healthcare costs from frequent hospital stays, tests, medications, and ongoing care. In Saudi Arabia, a recent 2023 study estimated that 77.6 children per million are receiving chronic dialysis, underscoring the significant national impact of this challenge [1].

Given that Saudi Arabia offers universal, government-funded healthcare, the financial responsibility for managing pediatric kidney disease, including dialysis, surgical interventions, and transplantation, rests primarily with the state [3]. Although dialysis is essential for survival, it is not a cure. When medically viable, kidney transplantation improves health outcomes and is less expensive than dialysis over the long term [4].

This research examines the economic implications of juvenile kidney disease and demonstrates how pediatric nephrology spending aligns with Saudi Vision 2030 goals for a healthier population and a more diverse, knowledge-based economy.

## Review

Literature identification and review approach

This is a narrative review of published studies on pediatric kidney disease in Saudi Arabian patients. The aim of this review is to examine the health costs and economic impact of dialysis and transplantation. The literature for this review was collected through a non-systematic search of peer-reviewed journals, national reports, and health economic studies on pediatric CKD, ESKD, dialysis, and kidney transplantation. Studies from Saudi Arabia were given priority, but international research was also used when local data were missing or when health systems were similar.

We were interested in studies on the prevalence of pediatric kidney disease, treatment success, healthcare utilization, the direct monetary cost of pediatric kidney disease, and its general socioeconomic implications. We included articles in English and Arabic. We excluded articles that focused solely on adult cases, animal models, or unrelated basic laboratory studies.

We summarized the findings to show the burden of pediatric kidney disease, compare the economic effects of dialysis and kidney transplantation, and assess how investing in pediatric nephrology can help support healthcare sustainability and the goals of Saudi Vision 2030.

The burden of pediatric kidney disease in Saudi Arabia is substantial, as children’s kidneys work differently from those of adults, which makes them more likely to develop congenital, hereditary, and genetic disorders. In Saudi Arabia, CAKUT is responsible for a large share of pediatric CKD cases [1]. When CKD develops in childhood, it can cause problems such as growth failure, cognitive issues, anemia, bone disease, and long-term health complications [1-4].

Besides CAKUT, several congenital and genetic kidney diseases also play a major role in pediatric CKD in Saudi Arabia. One example is congenital nephrotic syndrome, which usually appears in the first six months of life and needs intensive medical care to prevent it from progressing to ESKD. Nephrotic syndrome is more commonly diagnosed in toddlers and older children and is often related to genetic factors. Cystinosis is a rare inherited disorder that affects the kidneys and multiple other organs and may lead to progressive CKD. Recently, we identified a novel gene variant in Saudi Arabia that has not been previously registered in any global genetic database. This finding was documented and reported as a case report, expanding the current understanding of the genetic spectrum of this disorder [5]. Alport syndrome is another inherited disorder characterized by progressive kidney dysfunction, sensorineural hearing loss, and ocular abnormalities. In addition, lupus nephritis is an autoimmune kidney disease that occurs in children with systemic lupus erythematosus. Together, these conditions place a heavy burden on healthcare services and underscore the importance of specialized pediatric nephrology care. Recognizing these diseases early, making an accurate diagnosis, and managing them properly are key to reducing kidney damage and easing the social and economic impact.

Economic and social consequences of pediatric CKD are substantial. Pediatric CKD is associated with high healthcare utilization, such as frequent hospital admissions, dialysis sessions, and surgical procedures, which increases long-term costs. Furthermore, CKD-related fatigue, hospitalizations, and cognitive impairment all have a deleterious impact on academic performance. As a result, reduced future workforce readiness has been associated with childhood chronic illness, which has been linked to lower educational attainment and adult productivity. In addition, family burden is significant; parents may lose income due to caregiving responsibilities. Therefore, early diagnosis and timely intervention can prevent many of these complications, thereby reducing long-term socioeconomic consequences for both families and the national economy [6].

Prompt diagnosis and treatment of urinary tract infections, congenital anomalies, hypertension, and early CKD reduce complications, slow progression, and delay dialysis. Preventive care and early intervention are consistently more cost-effective than managing advanced CKD or ESKD. Children with stable kidney function have better school attendance and improved cognitive performance, which contribute to better long-term socioeconomic outcomes. Furthermore, socioeconomic studies support these observations by showing that childhood CKD, if not adequately managed, is associated with adverse adult financial and employment outcomes [7].

Economic impact of CKD in Saudi Arabia compared with dialysis and kidney transplantation highlights that kidney transplants are a more affordable and effective option than years of hemodialysis. Research shows they lower annual healthcare costs and support better long-term growth and development. Kidney transplantation is associated with fewer hospitalizations and significant improvements in quality of life when compared with long-term dialysis. A systematic review found that pediatric kidney transplants are more cost-effective than dialysis in many health systems [8]. Saudi Arabia already offers modern transplant services, and expanding pediatric transplant programs could further cut dialysis costs.

CKD, particularly end-stage renal disease (ESRD), places a substantial financial burden on Saudi Arabia’s healthcare system. Patients with ESRD require ongoing renal replacement therapy, most often hemodialysis, which involves frequent hospital visits and significant costs. In Saudi Arabia, each hemodialysis session costs approximately USD 297, totaling about USD 46,332 per patient annually [9].

A kidney transplant is more costly initially, but it is cost-saving in the end. Nevertheless, a medical investigation conducted in Saudi Arabia showed that dialysis is more costly than a transplant. It was found that the cost of dialysis would be USD 317,186 in four years, and the cost of a transplant is USD 210,779.

Other research supports these findings, showing that kidney transplantation helps people live longer and improves their quality of life, while also saving money over time compared to dialysis. The cost benefits are most noticeable after the first year, when ongoing care costs decrease [10].

This information is important for policymakers in Saudi Arabia as the country grows its national transplant programs and works toward Vision 2030 goals for better healthcare. Improving organ donation systems, increasing transplant numbers, and shortening wait times could make care more cost-effective, lower the economic impact of CKD, and improve long-term health for both children and adults.

**Figure 1:**
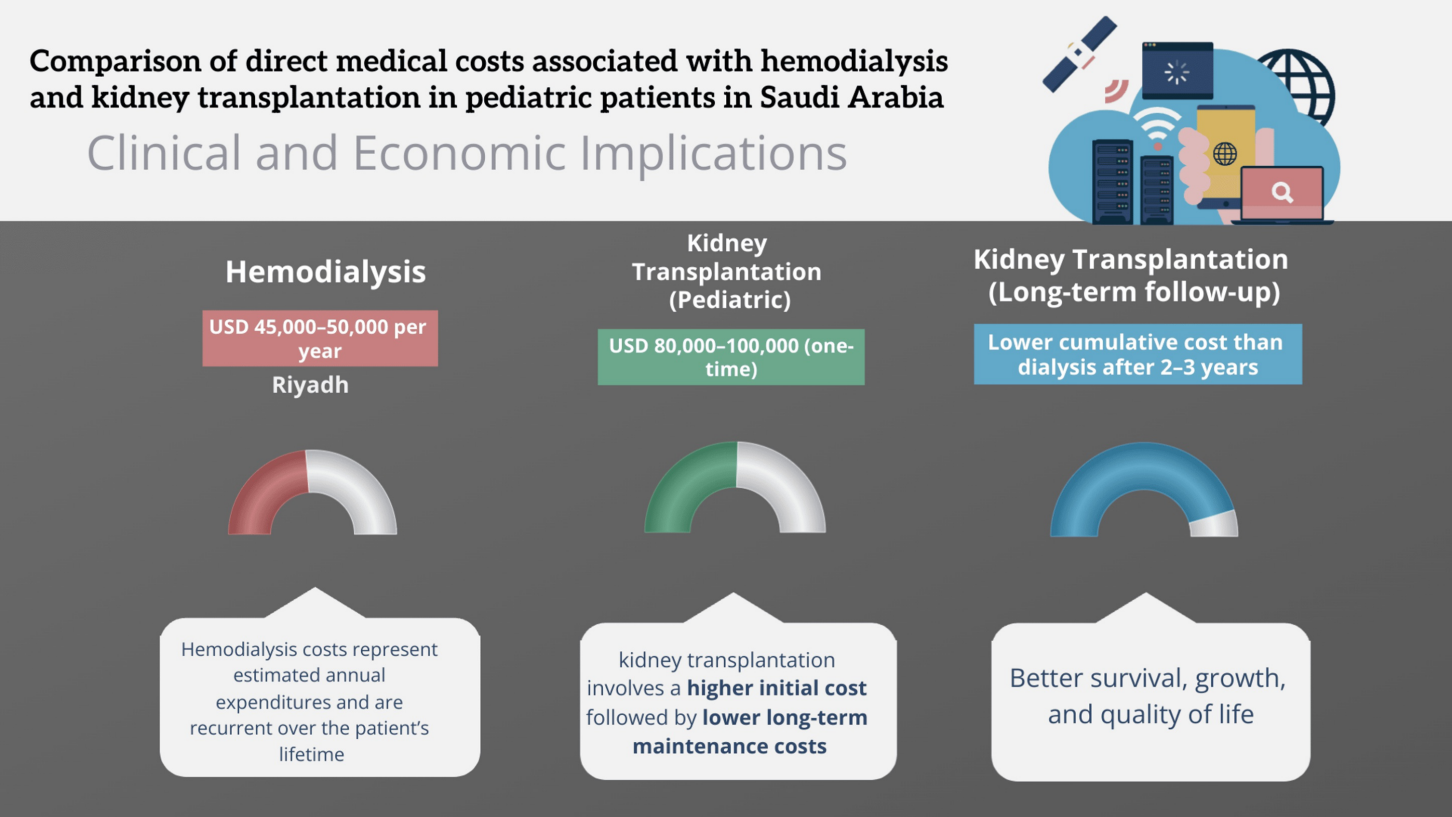
Comparison of cumulative long-term healthcare costs between pediatric hemodialysis and kidney transplantation in Saudi Arabia. References: [9,10] The figure was designed and generated by the authors using Visme (Visme.co), a licensed online design platform.

In pediatric patients with ESRD, hemodialysis is associated with substantial recurrent annual costs across major Saudi cities. Although kidney transplantation incurs higher initial expenditure, long-term follow-up demonstrates lower cumulative costs compared with continued dialysis after approximately two to three years, in addition to improved survival and quality of life outcomes as presented in Table 1 and shown in Figure 1 [9,10].

**Table 1:** Estimated direct medical costs and clinical implications of hemodialysis versus kidney transplantation in pediatric patients with end-stage kidney disease in Saudi Arabia based on published national and regional cost analyses. References: [9,10]

Treatment modality	Location / setting	Estimated cost	Cost horizon	Clinical and economic implications
Hemodialysis	Riyadh	USD 45,000–50,000 per year	Annual (recurrent)	High long-term cost due to lifelong treatment
Hemodialysis	Jeddah	USD 42,000–48,000 per year	Annual (recurrent)	Requires frequent hospital visits
Hemodialysis	Dammam	USD 40,000–46,000 per year	Annual (recurrent)	Significant cumulative financial burden
Hemodialysis	Other major cities	USD 38,000–45,000 per year	Annual (recurrent)	Does not include indirect societal costs
Kidney Transplantation (Pediatric)	National transplant centers	USD 80,000–100,000 (one-time)	Initial year	Higher upfront cost
Kidney Transplantation (Long-term follow-up)	Outpatient follow-up	Lower cumulative cost than dialysis after 2–3 years	Multi-year	Better survival, growth, and quality of life

Effective pediatric kidney care enables parents to return to work earlier, reduces absenteeism, and enhances household financial stability. Healthier children become productive adults, reducing future dependency on social and health services [7].

Cost optimization can also be achieved through the appropriate selection of dialysis modality. HD and PD are equally good options for treating children with ESKD. HD is usually performed in hospitals or dialysis centers and requires much more equipment, specialized personnel, and frequent hospital visits. By contrast, PD can be performed at home and requires less hospitalization, offering greater flexibility in treatment schedules. Peritoneal dialysis has some advantages over hemodialysis for children with ESKD. It can be done at home, so it requires less equipment and fewer specialized staff. This gives families more flexibility with treatment times and means fewer hospital visits. As a result, children can miss less school, and families can maintain more stable routines. Research shows that peritoneal dialysis can improve quality of life and lower healthcare costs, mainly because there are fewer hospital stays and less travel. For these reasons, choosing peritoneal dialysis first for eligible children in Saudi Arabia could be a cost-effective approach [11,12].

The role of pediatric nephrology in Saudi Arabia is central to the prevention and management of kidney disease in children. Pediatric nephrology involves diagnosing, treating, and regularly monitoring kidney problems in children. Improving these services helps the country achieve its health goals by finding kidney disease early, encouraging kidney transplants, lowering the need for dialysis, and helping children stay healthier in the long run. To get these results, it is important to focus on funding and building up expert care for children's kidney health in Saudi Arabia.

Key strategies for supporting national economic growth through pediatric nephrology, including value-based care, medical tourism, healthcare entrepreneurship, and research innovation, are summarized in Table 2 [3,6,8].

**Table 2:** Strategic priorities for advancing pediatric nephrology in Saudi Arabia and their potential contributions to national economic growth, synthesized from published health policy, economic, and pediatric nephrology frameworks relevant to Saudi Vision 2030. References; [3,6,8]

Strategy	Description
Enhancing National Productivity Through Improved Health	Improving the health of children with kidney disease supports the development of a healthier future workforce, reduces dependency, and increases national productivity.
Value-Based Care and Cost-Effective Treatment Pathways	Aligned with Saudi Vision 2030 health transformation goals, value-based pediatric nephrology includes PD-first approaches, standardized CKD management pathways, early transplant evaluation, and prevention-focused programs.
Medical Tourism and Regional Leadership	Establishing advanced pediatric nephrology and transplant centers positions Saudi Arabia as a regional hub for specialized care, attracting international patients and supporting economic diversification.
Healthcare Entrepreneurship and Local Industry Growth	Opportunities include the development of pediatric dialysis technologies, home-monitoring devices, nutrition and rehabilitation services, and pediatric-focused pharmaceuticals stimulating private-sector expansion and job creation.
Research and Innovation	Saudi Arabia can lead in genomic research of hereditary kidney diseases, innovative dialysis modalities, and pediatric transplant outcomes. Increased research productivity enhances global visibility and contributes to economic advancement.

Despite increasing recognition of the burden of pediatric kidney disease in Saudi Arabia, studies that comprehensively evaluate its economic impact in children remain limited. Although recent national and international data have begun to describe the direct medical costs of dialysis and transplantation in pediatric patients [13,14,15], important gaps persist in capturing the full economic burden of the disease. Indirect costs, including reduced caregiver productivity, disruption of children’s education, and long-term effects on workforce participation, are still insufficiently addressed in existing analyses. Expanding national pediatric kidney disease registries, standardizing cost-reporting methodologies, and incorporating economic evaluations into clinical outcome studies would provide more robust evidence to inform healthcare planning and policy decisions [14]. Strengthening collaboration between pediatric nephrologists, health economists, and policymakers is essential to optimize resource utilization and support the sustainable development of pediatric kidney care services in alignment with Saudi Vision 2030.

Strengths and limitations

The article provides a broad overview of kidney diseases in children in Saudi Arabia. It discusses problems that are present at birth, genetic disorders, and diseases caused by the immune system. It also discusses how frequently these diseases occur, their effects, and their costs.

The article also looks at the costs of dialysis, transplants, and long-term care, linking pediatric kidney care to national economic growth and the goals of Saudi Vision 2030. It suggests practical ways to improve care, such as value-based care, more organ transplants, medical tourism, entrepreneurship, and research support. The article connects clinical and economic issues to national healthcare policy to help guide decision-makers. This review uses a narrative approach, which may lead to selection bias and does not include a systematic meta-analysis of all available evidence. Although some regional studies are included, there is still a lack of nationwide, long-term data on outcomes and costs for pediatric kidney disease in Saudi Arabia. This makes it harder to apply the findings more broadly. Most cost-effectiveness estimates are based on published studies and models, which could change as healthcare policies and infrastructure develop. Finally, the review only covers pediatric kidney disease. It does not discuss adult kidney disease, related economic issues, or the transition from pediatric to adult care, all of which could affect long-term cost estimates.

## Conclusions

Pediatric nephrology brings important health and economic benefits to Saudi Arabia. Detecting kidney disease early, growing transplant programs, using cost-effective treatments, and supporting research are all key to lessening the impact of kidney disease in children.

To achieve these benefits and support Saudi Vision 2030, everyone involved should work together and act quickly. It is imperative to enhance early detection initiatives, increase investment in transplant infrastructure, expand financial support for pediatric kidney research, and raise public awareness.
